# Knowledge of Iranian Medical Interns Regarding Cardio-Pulmonary Resuscitation

**DOI:** 10.5812/traumamon.4230

**Published:** 2012-05-26

**Authors:** Hassan Ravari, Mojtaba Abrishami, Masoume Ghezel-Sofla, Mohammad Vahedian- Shahroodi, Mostafa Abrishami

**Affiliations:** 1 Vascular and Endovascular Surgery Research Center, Mashhad University of Medical Sciences, Mashhad, IR Iran; 2 Eye Research Center, Mashhad University of Medical Sciences, Mashhad, IR Iran; 3 Department of Health, Faculty of Paramedics and Health, Mashhad University of Medical Sciences, Mashhad, IR Iran; 4 Student Research Center, Mashhad University of Medical Sciences, Mashhad, IR Iran

**Keywords:** Cardiopulmonary Resuscitation, Emergency Medicine, Knowledge, Medical, Education

## Abstract

**Objectives:**

Cardio-Pulmonary Resuscitation (CPR) is one of the most important procedures in emergency medicine. As new trends are evolving in medical education , we planned to evaluate the interests and knowledge of medical students regarding educational methods in CPR learning.

**Materials and Methods:**

In a cross-sectional analytical descriptive study, a standardized questionnaire was distributed among 180 medical interns at the Mashhad University of Medical Sciences. The questionnaire had three parts: demographics, general questions, and CPR knowledge. If they had more than 10 correct answers (out of 15) in knowledge, they were placed in group A and if more than 5, in group B and correct answers less than 5 were categorized in group C.

**Results:**

159 interns filled the questionnaires. Mean age was 24.99 ± 0.96 and 56.5% were female; 52.7% were educated only theoretically and 47.3% had combined theoretical and clinical knowledge; male interns were significantly more educated (P = 0.041). Residents were the majority of trainers (56.8%) and only 14.3% were educated by the staffs. Only 7% mentioned that they felt they could do a complete CPR and 37.3% considered themselves as assistants; 93.7% believed that isolated emergency ward and teaching courses were needed for better education and 95% declared that continuous education is obligatory; 33.5% were in group A and 45.8% were in group B.

**Conclusions:**

CPR education is of interest to most interns. Due to lack of emergency medicine wards and, the interns’ knowledge and their practical skills were insufficient to perform acceptable CPR.

## 1. Background

Cardiac arrest is one of the most common causes of death nowadays. Early CPR is an efficient method to decrease the rate of death (1). The American Heart Association reports that deaths from out-of-hospital cardiac arrest can be significantly prevented if 20% of the population are trained for CPR. (2). Thus teaching CPR is really needed among the population as well as among medical students at healthcare centers.

CPR is an important skill for physicians in the field of Emergency Medicine. The methodology of teaching and learning methods for CPR is important. Amount of knowledge of participants was measured according to the last version of guidelines. Some wards such as anesthesia, internal medicine and cardiac wards optionally provided training at variable stages of medical training (when learning physiopathology or at externship (stager) or at internship). Certain organizations such as non-governmental organization (NGO) also teach CPR to students. We used a 3-page questionnaire which was filled without any difficulty by students. The questionnaire sought information about students’ interest, knowledge and skills of CPR. 

## 2. Objectives

There is a little data about formal training of CPR. As performing CPR is necessary for medical students, this study was done to seek valuable data about the knowledge, interest, desires and skills of interns in performing CPR.

## 3. Materials and Methods

This cross–sectional study was performed to measure interest and knowledge of medical students at internship level regarding CPR at the Imam Reza and Qaem hospitals, of the Mashhad University of Medical Sciences. Inclusion criteria to enter to the study were intern students studying medicine at the time of this study and their willingness to participate. One hundred eighty intern students entered the study.

Data was collected by using a standard questionnaire. This questionnaire consisted of 3 parts: demographic data, level of interest and general questions as well as 15 questions regarding CPR knowledge. If the correct answers were more than 10, they were categorized in group A, if the correct answers were more than 5, they were categorized in group B, and if the correct answers were less than 5, they were categorized in group C. The data was analyzed by SPSS software version 16.

## 4. Results

One hundred fifty nine interns filled and returned the questionnaire with a mean age of 24.99 ± 0.96 years ; 56.5% of participants were females and 43.5% were males. 52.7% were educated only theoretically and 47.3% had combined clinical and theoretical knowledge; male interns were significantly more educated (P = 0.041). Residents were the majority of trainers (56.8%) and only 14.3% were educated by faculty staffs. NGOs such as the Red Crescent Society or Basij were also ivolved in training (29%). Only 7% declared that they felt that they could do a complete CPR and 37.3% considered themselves as assistants; 93.7% believed that a specialized emergency ward and courses are needed for better education and 6.3% prefered optional training; 95% felt t continuous education is obligatory; 33.5% were in group A and 45.8% were in group B. Participants who thought that they could do CPR completely were all in group B. The internal medicine ward (30.8%) and neurology ward (27.1%) provided more practical instructions to CPR teams of intern students than other wards. The most theoretical information of CPR was given to students in the cardiology wards (38.9%).

## 5. Discussion

This study showed that a small percentage of interns believed that they knew how to carry-out CPR and felt that they can do it correctly (7% of all). It seems that without a specific ward for CPR training, interns may not acquire the confidence to correctly perform CPR. Moreover, without a curriculum, multiple departments believed that they were responsible for CPR education, and participated incompletely in this regard. It is obvious that this education is not enough as just 33.5% were only in group A. Even more so, on behalf of faculty members who had no obligation to train interns, residents (56.7%), not in classes, but at bedside provided this training. It is clear that interested students would participate in classes held by NGOs (29%). Most of the interns who were educated both theoretically and practically (47.3%) were trained by the NGOs (86.3%). It shows that less than half of the interns were trained both practically and theoretically, and in the absence of didactic education.

Interns themselves, believe that a separate department such as the emergency medicine department will help them in training. It is felt by the interns that without continuous education, CPR training is inadequate. Other studies show similar necessity to train different people for resuscitation activities to raise survival rates of patients’ arrest. Thorén et al. mentioned that the major cause of low trained rate of elderly people about CPR was less awareness about its benefits and availability. And also 96% percent of participants who took part in their study were willing to perform CPR by themselves if they needed to (2). Dane et al. showed that nurses who were trained for Advanced Cardiac Life Support (ACLS), diagnosed arrests more than non-trained nurses for ACLS (3).

Moretti et al. concluded that trained ACLS team members could increase long and short-term survival rates from cardiac arrest (1). Kahouei et al. stated that clinical experience is effective learning. A case in point in teaching hospitals is training medical students in emergency medicine. However, they believed that there is little information about medical students’ knowledge required to help them when they encounter emergency cases. In their study, Kahouei et al. aimed to survey the information needs of medical students during a clinical encounter. Evaluating 70 medical students as their participants, they found that most questions (42% of all questions asked by students) were about diagnosing the patients’ problems, and the least were about organizational procedures and hospital policies. The major information needed by the students were reported to be laboratories (84.3%) and radiography results (74.3%). Moreover, they mentioned that the few questions were asked about organizations and the interrelationship between them and clinical aspects of the job. They believed that organizational information and medico-legal issues should be taught to medical students to increase their awareness of these topics (4). Van der Vlugt conducted a study to measure the effect of medical students’ attendance in a course of preclinical emergency techniques and their medical knowledge as well as their technical skills. They found that students who had taken Essential Procedures in Emergency Medicine (EPEM) courses had higher medical knowledge and technical skills than those who had not. They also pointed-out that emergency medicine could be a suitable field to equip students with technical skills (5).

David et al. made an attempt to investigate the possibility of adding emergency medicine to the undergraduate medical curriculum. They recorded students’ feedback while training in emergency medicine, and concluded that emergency medicine is an absolute necessity for the undergraduate medical curriculum (6). Having given written examination on such skills, Kelly AM et al. evaluated recognition and managing critical situations among senior medical students in wards and compared those who had taken emergency medicine with those who had not. They found that students who had taken emergency medicine got better scores than those who had not (P < 0.001). Therefore, they suggested that studying emergency medicine could improve the skills of senior medical students in critical situations in wards (7). These studies were all based on the effective learning and training self-stemmed personnel and practitioners. Similarly our results showed the interest of medical students for CPR training and necessity of establishing a curriculum for CPR. Most recommended that a separate ward such as emergency medicine, should be established for students to learn CPR. Training for CPR is necessary for medical students to increase patient survival rates. Additionally, CPR education is one of the interests of most interns. A separate ward and curriculum are needed to better train for CPR and decrease deaths from cardiac arrests.
